# The epidemiology of sepsis: questioning our understanding of the role of race

**DOI:** 10.1186/s13054-015-1074-7

**Published:** 2015-10-01

**Authors:** Thomas S. Valley, Colin R. Cooke

**Affiliations:** Division of Pulmonary and Critical Care Medicine, Department of Internal Medicine, University of Michigan, Ann Arbor, MI 48109-2800 USA; Michigan Center for Integrative Research in Critical Care, University of Michigan, Ann Arbor, MI 48109-2800 USA; Center for Health Outcomes and Policy, University of Michigan, Ann Arbor, MI 48109-2800 USA; Institute for Healthcare Policy and Innovation, University of Michigan, Ann Arbor, MI 48109-2800 USA

## Abstract

Race has been identified as an important risk factor for the development of sepsis and as a predictor of poor outcomes in sepsis. For example, black individuals have been demonstrated to be nearly twice as likely to develop sepsis and to have greater mortality from sepsis than white individuals. Recent data from a longitudinal cohort, which examined incident hospitalizations for infections occurring among participants in the Reasons for Geographic and Racial Differences in Stroke (REGARDS) cohort, contradicts this prior research. Investigators determined that black participants were significantly less likely than white participants to present to the hospital with either infection or sepsis. Although these results are intriguing, they highlight our inadequate understanding of the relationship between race and sepsis and motivate the need for higher quality epidemiologic research to isolate the true role of race in the development of sepsis.

## Introduction

The study by Moore et al. [1], published in a recent issue of *Critical Care*, questions the school of thought that race is both an important risk factor for the development of sepsis (a common reason for hospitalization and present in nearly half of all hospital deaths [2]) and also as a predictor of sepsis outcomes [3]. For example, most studies suggest that black patients have nearly double the incidence rate of sepsis and greater sepsis-related mortality compared with white patients [4–6]. These racial disparities in sepsis have been attributed to inequalities in social determinants of health, pre-existing comorbidities, health behaviors, or access to high quality care [3, 5, 7, 8].

## Revisiting the role of race in sepsis

Moore and colleagues analyzed the Reasons for Geographic and Racial Differences in Stroke (REGARDS) cohort to characterize the incidence of hospital visits for infection or sepsis in black and white participants. Though designed as a large, prospective cohort to evaluate geographic and racial differences in risk factors for stroke, by enrolling community-dwelling individuals and following them for nearly 10 years, REGARDS allowed the investigators to examine whether participants’ hospital visits were due to infection or sepsis [9]. In addition, by identifying sepsis using medical record review, the investigators overcome the primary limitation of existing studies—the use of administrative data to detect sepsis. They demonstrate that, surprisingly, black participants were significantly less likely to present to the hospital with either infection or sepsis than white participants.

In contradicting more than a decade of existing epidemiologic research, the study by Moore et al. requires us to re-examine our understanding of race and sepsis risk. To do so, we must start by understanding why the study of Moore et al. differs from prior work. First, how could different methods of identifying sepsis across studies contribute to disparate sepsis rates? As the authors note, previous research used claims data to detect patients discharged from the hospital after treatment for sepsis [4–6]. While this method of identification has reasonable sensitivity and specificity, it may fail to account for variations in sepsis diagnosis coding between hospitals [10]. In other words, if hospitals that care for a higher proportion of black patients more readily include a code for sepsis in the discharge claim than other hospitals, the incidence rate of sepsis among black patients would be higher in studies using administrative data than on review of the medical record [11].

Second, could the timing or site where sepsis develops contribute to different estimates? Prior studies detected sepsis occurring at any time during a hospitalization and counted multiple episodes per patient [4–6], whereas Moore et al. examined participants’ first presentation to the hospital for infection or sepsis, discounting sepsis if recurrent or hospital-acquired. If black individuals have higher rates of recurrent or hospital-acquired sepsis than white individuals, this could explain the study’s departure from previously published research. Since black individuals are known to have higher odds of readmission after sepsis [12, 13] and face limited access to hospitals that provide higher quality of care [8], it is plausible that they may be more likely to experience a second episode of sepsis or be more prone to developing sepsis after presenting to worse hospitals for another condition.

Third, could differences in the sampling of patients across studies explain dissimilar sepsis rates? Several studies demonstrate age- and sex-specific differences in sepsis rates between black and white patients. For example, Mayr et al. [6] demonstrated that the most dramatic contrasts in sepsis rates between black and white patients occurred among those aged younger than 65 years. Similarly, Dombrovskiy et al. [3] and Martin et al. [4] found that the greatest differences between black and white patients in sepsis-specific hospitalization rates and mortality occurred in patients aged 35 to 44 years, particularly among males. Because the REGARDS registry included only those over the age of 45 years and disproportionately enrolled black women [9], it may have inadvertently selected a population in which black participants would be expected to have attenuated relative differences in rates of sepsis when compared with those in other studies.

## Conclusion

While it is intriguing to speculate about why the findings of Moore and colleagues differ from prior work, perhaps the most important question to consider is: what is the true relationship between race and sepsis risk? Prior to Moore et al., the answer was clear—black individuals experience a greater risk of sepsis than white individuals—yet now the answer is more elusive. Moore et al.’s findings highlight the flaws of conclusions based upon prior work and draw attention to the lack of sophistication in our understanding of how race influences the development and natural history of sepsis. To address these uncertainties and provide a definitive answer, a large, prospective cohort study focusing on the epidemiology of sepsis is necessary. Such a study could provide better insight into the role of race in sepsis, not only for black and white participants but for other understudied races as well. Only after we better understand the role of race in sepsis development will we be able to address the underlying causes that lead to such differences.

## Abbreviation

REGARDS: Reasons for Geographic and Racial Differences in Stroke
